# Cold- and hot-water immersion are not more effective than placebo for the recovery of physical performance and training adaptations in national level soccer players

**DOI:** 10.1007/s00421-025-05835-w

**Published:** 2025-06-11

**Authors:** Jannik Gustafsson, Diego Montiel-Rojas, Mattias G. A. Romare, Elin Johansson, Mattias Folkesson, Marco Pernigoni, Anastasija Frolova, Marius Brazaitis, Tomas Venckunas, Elodie Ponsot, Thomas Chaillou, Peter Edholm

**Affiliations:** 1 School of Health Sciences, Örebro University, Örebro, Sweden; 2 Department of Coaching Science, Lithuanian Sports University, Kaunas, Lithuania; 3 Institute of Sport Science and Innovations, Lithuanian Sports University, Kaunas, Lithuania; 4 Laboratory of Applied Biology, Research Unit in Applied Neurophysiology (LABNeuro), Faculty of Human Movement Sciences, Université Libre de Bruxelles, Brussels, Belgium; 5 Pain in Motion Research Group (PAIN), Department of Physiotherapy, Human Physiology and Anatomy, Faculty of Physical Education and Physiotherapy, Vrije Universiteit Brussel, Brussels, Belgium; 6 Institute of Metabolic and Cardiovascular Diseases, INSERM/Université de Toulouse, Team MetaDiab, Toulouse, France; 7 Department of Environmental and Bioscience, Halmstad University, Halmstad, Sweden

**Keywords:** Fatigue, Cooling, Heating, Football, Exercise, Athletes

## Abstract

**Purpose:**

Cold- and hot-water immersion (CWI and HWI, respectively) are popular post-exercise recovery methods in competitive soccer. The aims of this study were to (1) compare the effect of post-exercise CWI, HWI and placebo on the recovery of physical performance in national level soccer players, and (2) investigate whether repeated use of these recovery modalities has an impact on training adaptations over a 15 week period.

**Methods:**

For Part I, 40 male soccer players (15–19 years) were randomized to either CWI (10 °C, 10 min), HWI (42 °C, 20 min), or placebo (6 min, sham laser), applied after a 90 min simulated soccer match (SSM). Physical performance was assessed using submaximal aerobic, 20 m sprint, countermovement jump (CMJ), and knee extension strength tests [i.e., maximum voluntary isometric contraction (MVIC) and time to exhaustion (TTE) at 60% of MVIC] performed at Pre-SSM and 0, 21 and 45 h Post-SSM**.** For Part II, 19 participants applied their respective recovery modality (~ twice a week) in their usual training. After 15 weeks, physical performance and body composition were assessed and compared to pre-intervention**.**

**Results:**

All three modalities similarly affected the recovery of physical performance during the 21–45 h Post-SSM period (p < 0.05). Moreover, no significant effects of the recovery modalities on body composition and on development of physical performance were found over the 15 week recovery intervention (p > 0.05).

**Conclusion:**

Compared to a placebo, CWI and HWI do not improve post-match recovery of physical performance and do not impact long-term training adaptations in highly trained soccer players.

**Supplementary Information:**

The online version contains supplementary material available at 10.1007/s00421-025-05835-w.

## Introduction

Competitive soccer is an intermittent sport involving repeated high-intensity efforts, including sprints, jumps, changes of direction, as well as submaximal running. These demands, combined with congested schedules over the season requires optimizing recovery strategies (Nédélec et al. 2012). In addition to reducing injury risks, these strategies can also be beneficial to enhance the restoration of physical performance, allowing for increased training load, which ultimately can maximize training adaptations (Nédélec et al. 2012).

Among the recovery modalities utilized in soccer, cold-water immersion (CWI) is commonly applied by most elite soccer teams (Altarriba-Bartes et al. 2021). However, the popularity of CWI is generally based on the belief in the modality rather than on strong scientific evidence of its efficacy to enhance the recovery of physical performance (Broatch et al. 2014). Studies examining the effects of post-exercise CWI on recovery in soccer players yield conflicting findings. While some results indicate benefits of CWI on recovery of physical performance (e.g. jump, sprint, strength and running) (Ascensão et al. 2011; Bouchiba et al. 2022; Bouzid et al. 2018; Pooley et al. 2020; Rowsell et al. 2011), others do not (Coelho et al. 2021; Nasser et al. 2023; Rowsell et al. 2009; Rupp et al. 2012). Such discrepancies could be due to varying characteristics among study populations (e.g. age and competitive level) and the nature of the protocol replicating soccer-specific physical demands (i.e. match vs. intermittent running). It had previously been hypothesized by Versey and co-workers (Versey et al. 2013), that repeated use of CWI could enhance the development of physical performance. However, a recent meta-analysis indicates that it could negatively impact resistance training adaptations, without affecting endurance training adaptations (Malta et al. 2021). Specifically, the development of maximal strength, fatigue resistance, and jump performance, as well as muscle hypertrophy were impaired in active men when CWI was applied after resistance exercise sessions (Fröhlich et al. 2014; Fyfe et al. 2019; Roberts et al. 2015). These impairments may be attributed to the negative effect of post-exercise CWI on protein synthesis, and anabolic signaling (Betz et al. 2025; Fuchs et al. 2020; Fyfe et al. 2019; Roberts et al. 2015), which have been shown to attenuate increases in skeletal muscle mass (Roberts et al. 2015). In soccer, which combines aerobic-demanding exercises with high-intensity efforts, CWI might negatively affect the development of strength-related physical performance. This may be of particular importance in the context of young highly trained players that are still developing physically.

In contrast, post-exercise hot-water immersion (HWI), is less frequently utilized compared to CWI as a recovery modality among athletes and elite soccer teams (Altarriba-Bartes et al. 2021). The use of HWI has recently gained interest among scientists (McGorm et al. 2018). Despite limited research, emerging literature report similar benefits to that of previous CWI studies, including improved recovery of jump power (Viitasalo et al. 1995). Notably, when compared to CWI, HWI applied between two daily training sessions has shown the potential to improve repeated sprint performance in elite ice skaters (Solsona et al. 2023) and endurance fatigue resistance in young active adults (Cheng et al. 2017), respectively. These improvements may result from changes in intramuscular temperature, which subsequently may enhance muscle glycogen resynthesis (Cheng et al. 2017; Solsona et al. 2023). In contrast to CWI, repeated HWI can enhance the development of maximal isometric strength in elite track speed skaters (Méline et al. 2021), but not jump performance (Horgan et al. 2023; Méline et al. 2021) or lean mass (Horgan et al. 2023). Some evidence from animal studies suggests that HWI may improve muscle regeneration after injury (McGorm et al. 2018). Post-exercise HWI could be incorporated into regular training to enhance training adaptations (Thorpe 2021). However, whether the incorporation of HWI may affect recovery from training and/or matches in soccer players is currently unknown and warrants investigation (Thorpe 2021). While yet to be determined, HWI may promote the recovery of physical performance and enhance long-term training adaptations in soccer players.

Recovery is not solely a physiological process. The placebo effect, a psychobiological response known to affect exercise performance, is an important factor that should not be overlooked (Beedie and Foad 2009). The benefits of recovery strategies such as CWI and HWI can be attributed to an enhanced perceptual outcome as well as an athlete’s belief in the effectiveness of the recovery modality. Numerous studies have shown that CWI following different forms of exercise is not more effective than placebo for improving recovery (Broatch et al. 2014; Nasser et al. 2023; Wilson et al. 2018, 2019, 2021). In contrast to previous recovery studies within soccer (Ascensão et al. 2011; Bouzid et al. 2018; Coelho et al. 2021; Pooley et al. 2020; Rowsell et al. 2011, 2009; Rupp et al. 2012), to our knowledge, only one study included a placebo condition, showing that benefits of CWI and placebo on recovery of physical performance were similar (Nasser et al. 2023). In addition, a recent study, comparing repeated applications of placebo and CWI following resistance exercise, did not find any differences between these modalities after 8 weeks with regard to strength and perceptual benefits in untrained men (Wilson et al. 2021). To date, there are no studies comparing the recovery effect and training adaptations between HWI, CWI and placebo in soccer players.

In this study, the first aim was to compare the effect of post-exercise CWI, HWI and placebo on the recovery of physical performance in national level youth soccer players. The second aim was to investigate whether the repeated use of these post-exercise recovery modalities has an impact on training adaptations over a 15 week period.

We hypothesized that CWI and HWI are not more effective than a placebo treatment for recovery following a simulated soccer game. In longer term perspective, when regularly applied after intense soccer training over a 15 week period, HWI may enhance the development of physical performance, whereas CWI could have a detrimental effect.

## Materials and methods

### Participants

Forty-three male adolescent soccer players were recruited from the academy of a local professional club. All three of the academy teams (u-16, u-17 and u-19) were competing in the highest Swedish league, and a few participants were part of the Swedish youth national team. Participants trained ~ 5 sessions per week, including ~ 1 competitive match. Training consisted of 2–3 intense sessions (60–90 min) and one moderate intensity session (60 min). Furthermore, one session was followed by whole body strength exercises (30–45 min). For the present study, only field players were eligible for inclusion, given that they were (1) healthy and injury-free, (2) competitively active soccer players and not consuming any banned ergogenic substances or doping-classified medication. One participant regularly used prescribed asthma medication, and another prescribed immuno-suppressive medication. Both were included as they were deemed healthy by the club’s medical team. Three participants dropped out during baseline testing (day 1 of part I) due to musculoskeletal pain (*n* = 2) and dizziness (*n* = 1). Hence, a total of 40 subjects were included in the final analyses of Part I of the study. The participants’ characteristics are presented in Table 1. Of these 40 participants, a subset of 19 participants were included in Part II (15-week recovery intervention). This large difference was due to the following reasons: called up to the professional squad (n = 1), injured (n = 1), stopped playing soccer (n = 3), unable to retain their place in the team due to academy structural reorganization (n = 15), poor compliance during intervention (n = 1). All participants provided written informed consent after having received a thorough explanation of the study procedures. The study protocol was approved by the Swedish Ethical Review Board (Dnr 2021–03148) and was in agreement with the Declaration of Helsinki and all national and regional regulations.

**Table 1:** Participants’ characteristics at baseline and load assessment during SSM (Part I)

		Placebo *N* = *13*	CWI *N* = *13*	HWI *N* = *14*
Participants’ characteristics	Age (yr)	17.1 ± 1.2	17.3 ± 1.1	17.2 ± 1.2
	Height (cm)	177.4 ± 6.9*	184.0 ± 5.6	178.2 ± 4.3*
	Body mass (kg)	68.0 ± 8.4	69.2 ± 4.5	68.8 ± 7.6
	Body fat percentage (%)	16.2 ± 3.0	15.1 ± 2.7	16.2 ± 3.4
	Fat free mass (kg)	56.9 ± 6.1	58.8 ± 4.5	57.6 ± 5.6
Neuromuscular performance	20 m sprint time (s)	3.15 ± 0.11	3.11 ± 0.13	3.08 ± 0.21
	CMJ height (cm)	29.9 ± 2.7	32.9 ± 3.1	32.7 ± 4.7
	MVIC peak force (N)	613 ± 90	655 ± 107	670 ± 112
Load assessment during SSM	Total distance (m)	10 839 ± 921	10 399 ± 502	10 429 ± 673
	HR average (bpm)	160 ± 12	163 ± 10	167 ± 12
	RPE at 90 min (1–10)	8.9 ± 1.3	8.6 ± 1.0	9.1 ± 0.7
	Lactate at 90 min (mM)	3.7 ± 1.5	3.3 ± 1.3	4.1 ± 2.8

Data are presented as mean ± SD. * p < 0.05 vs CWI

*CMJ* countermovement jump, *CWI* cold-water immersion, *HR* heart rate, *HWI* hot-water immersion *MVIC* maximal voluntary isometric contraction of knee extension, *RPE* rating of perceived exertion *SSM* simulated soccer match

### Experimental design

**Fig. 1:**
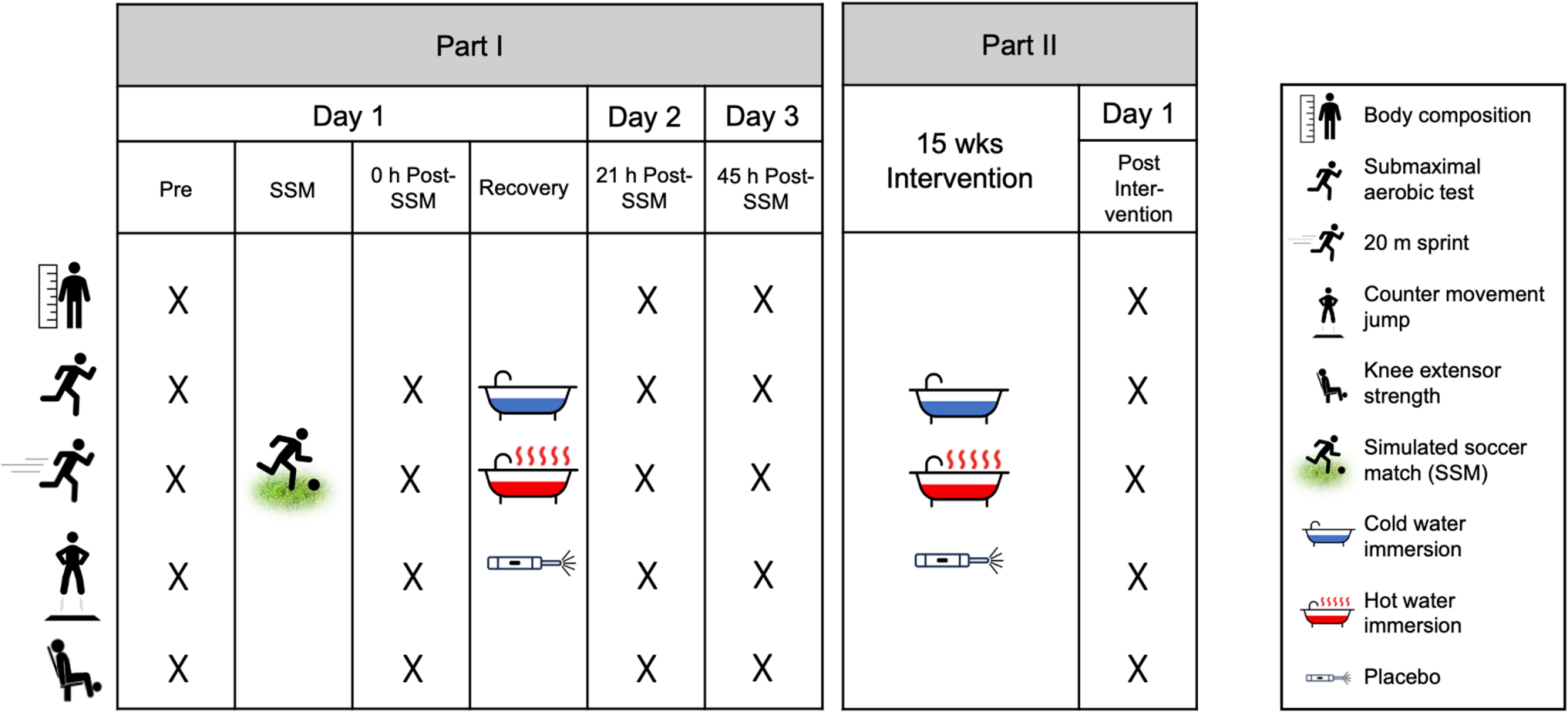
Experimental design

An overview of the study procedures is shown in Fig. 1. The study was composed of two parts. Part I investigated the effect of a single exposure of CWI and HWI and placebo following a simulated soccer match (SSM) on the recovery of physical performance at 21- and 45-h after the SSM. Part II consisted of a 15 week recovery intervention. The study followed a three-group randomized controlled design where participants were stratified according to team (u16, u17, u19) before being randomized to either a CWI, HWI or placebo group.

#### Part I: functional recovery following a simulated soccer match

Part I consisted of three days during a period from mid-October to mid-November 2021. Each participant arrived at the laboratory at the same time all mornings (7:00–10:30) in a fasted state. Directly after arrival, participants’ body composition [height and bioelectrical impedance analysis] was assessed prior to consuming a standardized breakfast. Thirty minutes after breakfast, participants undertook a battery of physical performance tests lasting 30–40 min consisting of a submaximal aerobic test, 20 m sprint test, countermovement jump (CMJ) and strength tests to establish baseline (Pre) values. Then, participants performed a 90-min SSM, after which they once again executed the same battery of tests to assess the direct impact of the SSM on physical performance (0 h Post-SSM). Immediately after the second battery of physical performance tests, the participants undertook their respective recovery modalities (CWI, HWI or placebo), followed by a standardized lunch and rest (2 h total). The following two days, participants underwent the same experimental procedures as before the SSM on day 1 (body composition, breakfast, battery of physical performance tests) to assess recovery. For each participant, the battery of physical performance tests was performed at the same time each day (in order to evaluate recovery of physical performance 24 h and 48 h after Pre-SSM/baseline assessment), meaning that on day 2 and day 3, performance was assessed 21 h and 45 h Post-SSM, respectively.

Of note, biological samples (muscle micro-biopsies, saliva, and venous blood) were taken on two occasions on day 1 (in the morning before breakfast and in the afternoon following the meal and resting period) as well as on day 2 (in the morning before breakfast) and on day 3 (in the morning before breakfast; only saliva and venous blood samples). The biological samples were collected for additional analyses focusing on biological responses to the recovery modalities, not included in this study.

#### Part II: 15-week recovery intervention

Part II of the study commenced early January 2022, after the Christmas break. Over an uninterrupted period of 15-weeks, participants returned to their normal training and match schedule of their respective teams as described above (*2.1 Participants*). During this period, they were planned to perform their respective recovery modalities (as assigned during the Part I of the study) after the intense training sessions (2–3 times per week). After this period, they underwent the same standardized procedures and testing protocol as those in Part I before the SSM (Pre-SSM).

#### Familiarization, standardization, and general conditions of the experimental design

Participants undertook familiarization trials of the battery of physical performance tests and the SSM at least one week before Part I. Starting one day prior, and throughout the whole duration of Part I and the post-intervention period of Part II, participants were provided with standardized meals. Participants were encouraged to follow their regular diet during the 15 week recovery intervention. The standardized meals were developed using the software Dietist Net Pro (Kostkoll AB, Bromma, Sweden). To meet the recommendations for daily recovery after training and match in soccer players (> 6 g/body mass carbohydrate and > 1.6 g/body mass protein) (Collins et al. 2021), total energy intake was defined according to three body mass categories (< 65, 65–80, > 80 kg). Twenty-five percent of the total energy intake was from fat, and the meals included a variety of bread, cereals, pasta/rice, meat, milk/sour milk, energy bars, fruit, and vegetables to ensure adequate intake of macro- and micronutrients. In addition, participants consumed a 700 ml sports drink containing 60 g of carbohydrates (Enervit, Milano, Italy) during the first half of the SSM. Water intake was allowed ad libitum. The participants were instructed to eat their last meal before 9 pm prior to each experimental day after which only water intake was allowed. For Part I, participants and researchers were blinded as to the assigned recovery modality of each subject throughout the Pre-SSM (baseline) tests and during the SSM as well as the 0 h Post-SSM tests. Participants were informed about their assigned recovery modality upon arriving at the recovery room, ~ 5 min before starting their assigned recovery modality. For Part II, neither the participants nor the researchers/evaluators were blinded to the recovery modality.

### Battery of physical performance tests

#### Submaximal aerobic test

First, a moderate-intensity submaximal aerobic test was performed based on the Yo-Yo Intermittent Recovery Test 1. During the test, participants ran for a duration of 4 min until they reached level 13.4 (i.e. total distance of 440 m). The test was designed to induce a substantial increase in heart rate without generating excessive fatigue. Heart rate (HR) was measured using a HR monitor (Vantage V2 or M400, Polar Electro, Bromma, Sweden) and the final value was recorded. Rating of perceived exertion (RPE, 1–10 scale) was assessed at the end of the test, and capillary blood lactate concentration (bLac) was analyzed using the Lactate Plus meter (Nova Biomedical, USA) within the first minute after completion. The test was conducted indoors on an artificial surface.

#### 20 m sprint

Three minutes after the submaximal aerobic test (which also served as an incremental warm-up before the sprints), two maximal 20 m sprints were performed with 2 min rest between. Two wireless photocell timing gates (MuscleLab, Ergotest, Langesund, Norway) were positioned at 0 and 20 m. Participants wore running shoes. The sprints were initiated using a falling start from 0.7 m behind the starting line. The faster of the two 20 m sprint times was selected for subsequent analysis. The test was conducted indoors on an artificial surface.

#### Countermovement jump (CMJ)

After the running tests, participants moved to the laboratory to perform the CMJ test. This test was executed ~ 5 min following the 20 m sprint test and consisted of three attempts separated by 30 s of rest. For each attempt, participants were instructed to jump as high and as fast as possible. Hands were positioned on the hips throughout the jump, and squat depth was self-selected. Jumps were visually validated, and non-compliant trials were redone. Kinetic data were collected at 100 Hz from the vertical axis using a 3D force platform (Kistler, 9281B, Kistler Nordic AB, Jonesered, Sweden) using LabJack U12 software (LabJack Corporation, USA). Raw vertical ground reaction force (GRF) data were analyzed unfiltered via an adapted custom Matlab script (r2021a, The MathWorks, Inc., Natick, MA) (Harry 2021). CMJ phases and selected variables [i.e. jump height (cm), squat depth (cm), absolute and relative peak force (N, N/kg) and peak power (W, W/kg)], were identified as previously described (Harry 2021). Jump height was calculated using velocity at takeoff. The attempt yielding the highest jump height was selected for subsequent analysis.

#### Strength tests of the knee extensors

Isometric strength tests were performed in the laboratory directly following the CMJ test. Absolute and relative maximal voluntary isometric contraction (MVIC) peak force (N, N/kg) was assessed by knee-extensor MVICs of the dominant leg using a force sensor (MuscleLab, Force Gauge ± 300 kg, Ergotest Innovation AS, Norway) attached to a knee extension machine (Nordic Gym, Sweden). The protocol was adapted from another study (Place et al. 2007). The machine was individually adjusted to the anthropometrical characteristics of the participants, who were seated, with their knees positioned at an angle of 80–90° and their hips at an angle of 90–100°. Straps were used to secure the chest, hip, and the non-dominant leg, and participants were instructed to generate maximal force. Participants performed three 5 s MVICs with a 1 min rest period between trials. The highest recorded value was selected for analysis.

Knee extension time to exhaustion (TTE) at 60% of the MVIC peak force was performed using the same settings as the MVIC test. Participants received verbal encouragement and visual feedback from a computer screen. The attempt was ended upon volitional fatigue or when the participant dropped below 50% MVIC. During the subsequent analysis, the point of failure was set at 55% MVIC force given that 60% MVIC force was not reattained within the next 3 s. TTE was calculated using a custom Matlab script (r2021a, The MathWorks, Inc., Natick, MA). Post-intervention TTE (Part II) was performed using 60% of pre-intervention MVIC peak force.

These physical performance tests (20 m sprint, CMJ test, and MVIC test) were selected as they are widely used and exhibit high reliability in elite athletes across a variety of sports (Edholm et al. 2014; Ferguson et al. 2024; Fristrup et al. 2024; Nasser et al. 2023; Warr et al. 2020).

### Simulated soccer match (SSM)

The SSM was modified from the original Copenhagen Soccer Test, validated for elite adult players (Bendiksen et al. 2012), to be suitable for adolescent players. The whole 90 min SSM was piloted in other adolescent soccer players of comparable level. As in the Copenhagen Soccer Test, our SSM consisted of two 45 min periods with 15 min of rest in between, and a similar course layout. The modifications included changing the 30 m airborne pass to an 8 m hard, airborne pass. Running speeds were presented to the participants as percentages of maximal speed, with 100, 80, 65, 50, and 40% corresponding to full, high, moderate, low speed and jogging, respectively. Participants carried out the light and moderate laps defined in the original protocol in an alternating order, with 20 s rest between each lap. Each participant was assigned one experimenter that supervised, instructed and provided ample verbal encouragement throughout the entire SSM to facilitate adherence to the protocol. The SSM was undertaken outside on a natural grass pitch with an ambient temperature of 8.7 ± 3.6 °C (−0.2 to 17.1 °C) and ambient humidity of 68.0 ± 15.6% ranging from (26–93%). Distance covered during the game was calculated based on the course layout. HR was continuously recorded. RPE and bLac were individually assessed within the first min after the second half.

### Recovery modalities

Participants were randomized to either a CWI (10 °C, 10 min), HWI (42 °C, 20 min), or placebo (6 min, sham laser) group. During Part I, the recovery modalities were undertaken directly after the second physical performance test battery (~ 45 min after SSM). Participants of the HWI and CWI group were seated and immersed in a bath up to the iliac crest. Ten min CWI at 10 °C is a commonly used protocol (Ascensão et al. 2011; Bouchiba et al. 2022; Bouzid et al. 2018; Wilson et al. 2021) while HWI immersion of at least 20 min (~ 41 °C) has been shown to be effective for improving post-exercise recovery (Sautillet et al. 2023; Solsona et al. 2023). Water temperature was set by draining water and/or adding warm water, cold water and/or ice on top, and was monitored throughout the whole immersion duration using a digital thermometer. The placebo condition consisted of sham laser treatment. Briefly, a sham device that had no functioning emitting laser was applied in accordance to a standardized protocol. The sham laser was applied to the proximal, middle and distal aspect of the anterior part of the thigh (1 min), followed by the proximal, middle and distal aspect of the lateral part of each thigh (1 min). The procedure began with the dominant leg followed by the non-dominant leg while participants were lying supine on a massage bed. Thereafter, with participants lying prone on the massage bed, the “laser” was applied to the proximal, middle and distal part of the posterior thigh of the dominant and non-dominant leg. All three conditions were undertaken at room temperature (~ 20 °C). All participants were told that each of the three recovery modalities had been proven effective, although it remained unclear which modality was the most effective.

The 15 week period of Part II of the study was undertaken between January and May, and training sessions took place outdoors on an artificial grass field (ambient temperature of 5.4 ± 5.6 °C and humidity of 42.8 ± 14.9%). The protocol of the 3 recovery conditions (CWI, HWI and placebo) was similar as during Part I, with the exception of being undertaken directly after a training session ($\sim$ 5 min post training) and using 6-seater M-Spa Rimba Urban baths for CWI and HWI (M-SPA, Kungälv, Sweden). The CWI and HWI took place in the shower room (~ 20 °C) while the laser treatment was in a separate room (~ 20 °C). All recovery treatments, performed by trained staff, were undertaken immediately after a quick (~ 30 s) shower, and participants were instructed to refrain from showering afterwards. Participants took turns for the sham laser, with treatment being between 5 and 20 min after training.

### Statistical analysis

Data are presented as mean ± standard deviation (SD). Statistical analyses were performed using GraphPad Prism (Graphpad Prism 10.2.1, San Diego, CA, USA), and the α-level of significance was set at p < 0.05. Normality of residuals was checked visually with QQ plots. When applicable, Geisser-Greenhouse corrections were used to correct for violation of the sphericity assumption. A one-way analysis of variance (ANOVA) was used to assess 1) the groups’ characteristics and physical performance at Pre, 2) loads during the SSM, and 3) number of training and recovery sessions in Part II. For RPE, data underwent aligned rank transformation (ART, ARTool for Windows, version 2.1.2; Feys 2016) before being analyzed as the other variables. A priori sample size calculation (α = 0.05, power = 0.8, effect size f = 0.25) estimated that at least 30 participants were required. To account for potential dropouts (20%), we aimed to recruit 45 participants (15 per group). This estimation was further supported by prior studies using comparable outcomes and methodologies (Broatch et al. 2014; Vieira et al. 2016; Bouzid et al. 2018). For Part II of the study, all available participants were included.

For Part I, mixed-effects models were first used with a three levels between-group factor (i.e., placebo, CWI and HWI) and a two levels within-group factor (Pre-SSM and Post-SSM) to check that changes in the dependent variables were similar between groups at Post-SSM. Thereafter, mixed-effects models with a three levels between-group factor (i.e., placebo, CWI and HWI) and three levels within-group factor (Pre-SSM, 21 h Post-SSM, and 45 h Post-SSM) were used to (1) assess the recovery pattern of the dependent variables and (2) determine whether the recovery modalities specifically affect their restoration. Subject were treated as random factors. When a time effect was observed, Dunnett’s multiple comparisons test was used to compare the other two time points (21 h and 45 h Post-SSM) to Pre-SSM across the three groups. If any interaction was detected, a Bonferroni’s multiple comparisons test was used to compare the groups. Mixed-effects models were used to account for missing data.

For Part II, mixed ANOVA was used with a three levels between-group factor (i.e., placebo, CWI and HWI) and a two levels within-group factor (Part 1, day 1 Pre- and Post-intervention). When an interaction was detected, a Bonferroni’s multiple comparisons test was used to compare the Pre- and Post-intervention time points. Due to low sample size and technical issues, HR at the end of the submaximal aerobic test in the CWI group was not included in the analysis of Part II.

## Results

### Participants’ characteristics at Pre, load assessment during SSM, and physical performance at Pre

No differences were observed for the participants’ characteristics in Part I at Pre (n = 40), except for height, where the CWI group was taller than the placebo and HWI groups (p < 0.05; Table 1). The analysis of the SSM did not reveal any differences between the groups for distance covered, average HR, RPE and bLac at the end of 90 min (Table 1). No differences between the three groups were found for the battery of physical performance tests at Pre (data not shown). For Part II (n = 19), no differences were observed for the participants’ characteristics and the battery of physical performance tests at Pre-Intervention (Table 2). The only exception was for age, where the PLA group was younger compared to the CWI group (p < 0.05) (PLA: 16.3 ± 0.8; CWI: 17.4 ± 0.6; HWI: 16.6 ± 0.9 years).

**Table 2:** Height, body composition and performance assessments before and after the 15 week recovery intervention (Part II)

		Placebo (n = 6)		CWI (n = 7)		HWI (n = 6)		Pre—Post intervention effect	Group effect	Interaction
		Pre	Post	Pre	Post	Pre	Post			
Height and Body Composition	Height (cm)	178.3 ± 6.7	179.1 ± 7.0*	183.4 ± 5.1	183.9 ± 4.9	177.9 ± 1.6	179.3 ± 2.0*	** < 0.001**	0.140	**0.022**
	Body mass (kg)	68.9 ± 7.3	71.3 ± 7.9	66.6 ± 3.6	68.7 ± 6.1	68.4 ± 7.2	70.5 ± 6.1	** < 0.001**	0.747	0.912
	Fat-free mass (kg)	57.2 ± 5.1	59.1 ± 6.1	57.2 ± 3.7	59.1 ± 3.4	56.5 ± 4.8	58.5 ± 4.3	** < 0.001**	0.959	0.991
	Body fat (%)	16.9 ± 2.7	17.1 ± 2.7	14.1 ± 1.9	13.9 ± 2.1	17.2 ± 2.3	16.9 ± 2.0	0.781	**0.036**	0.733
Submaximal aerobic test	HR (bpm) ^†^	178 ± 16	174 ± 14	/	/	170 ± 22	162 ± 16	0.107	0.411	0.564
	RPE (1–10)	6.7 ± 1.2	6.7 ± 1.0	5.6 ± 0.8	5.9 ± 0.9	6.7 ± 1.2	6.7 ± 0.5	0.301	0.102	0.990
	Lactate concentration (mM)	4.5 ± 1.7	4.4 ± 1.1	3.8 ± 0.8	4.5 ± 1.4	4.4 ± 2.0	4.1 ± 2.1	0.844	0.925	0.512
Sprint	20 m sprint time (s)	3.15 ± 0.12	3.08 ± 0.12	3.14 ± 0.14	3.15 ± 0.09	3.20 ± 0.23	3.16 ± 0.22	**0.043**	0.809	0.066
CMJ ^‡^	Height (cm)	29.6 ± 2.5	29.1 ± 3.0	32.9 ± 2.0	31.2 ± 4.0	30.6 ± 4.1	29.6 ± 2.9	**0.014**	0.328	0.445
	Peak force (N)	1525 ± 175	1684 ± 163	1543 ± 150	1643 ± 173	1642 ± 310	1770 ± 332	**0.004**	0.612	0.825
	Relative peak force (N/kg)	22.1 ± 1.2	23.7 ± 1.6	23.0 ± 1.9	23.9 ± 2.4	23.9 ± 2.8	25.1 ± 3.9	**0.043**	0.457	0.884
	Power (W)	3395 ± 297	3466 ± 290	3572 ± 474	3560 ± 551	3470 ± 437	3583 ± 551	0.119	0.861	0.350
	Relative power (W/kg)	49.4 ± 3.8	48.8 ± 3.5	53.2 ± 4.4	51.6 ± 5.2	50.8 ± 3.9	50.8 ± 6.3	0.144	0.470	0.416
	Squat depth (cm)	32.0 ± 3.2	31.0 ± 4.1	37.0 ± 3.7	33.3 ± 4.8	32.0 ± 7.1	30.1 ± 7.6	0.059	0.298	0.589
MVIC	Peak force (N) ^§^	614 ± 62	759 ± 62	631 ± 102	696 ± 77	629 ± 87	738 ± 89	** < 0.001**	0.848	0.198
	Relative peak force (N/kg)	9.0 ± 0.7	10.6 ± 0.6	9.5 ± 1.5	10.1 ± 1.0	9.2 ± 0.9	10.5 ± 1.3	** < 0.001**	0.994	0.173
	TTE (s) ^¶^	36.3 ± 8.8	39.3 ± 6.6	28.4 ± 7.3	31.5 ± 3.9	29.3 ± 5.4	42.5 ± 9.7	**0.021**	0.073	0.199

Data are presented as mean ± SD

*CMJ* countermovement jump, *CWI* cold-water immersion, *HWI* hot-water immersion, *MVIC* maximal voluntary isometric contraction of knee extension, *RPE* rating of perceived exertion, *TTE* time to exhaustion

^†^: HR not included in analysis of Submaximal aerobic test for CWI; ^‡^: Placebo, n = 6; CWI, n = 6; HWI, n = 6; ^§^: Placebo, n = 5; CWI, n = 7; HWI, n = 6; ^¶^: Placebo, n = 5; CWI, n = 6; HWI, n = 5; Bold values indicate *p* < 0.05; * p < 0.05 vs Pre Intervention

### Part I: Acute response to a single recovery session following the SSM

#### Submaximal aerobic test

**Fig. 2:**
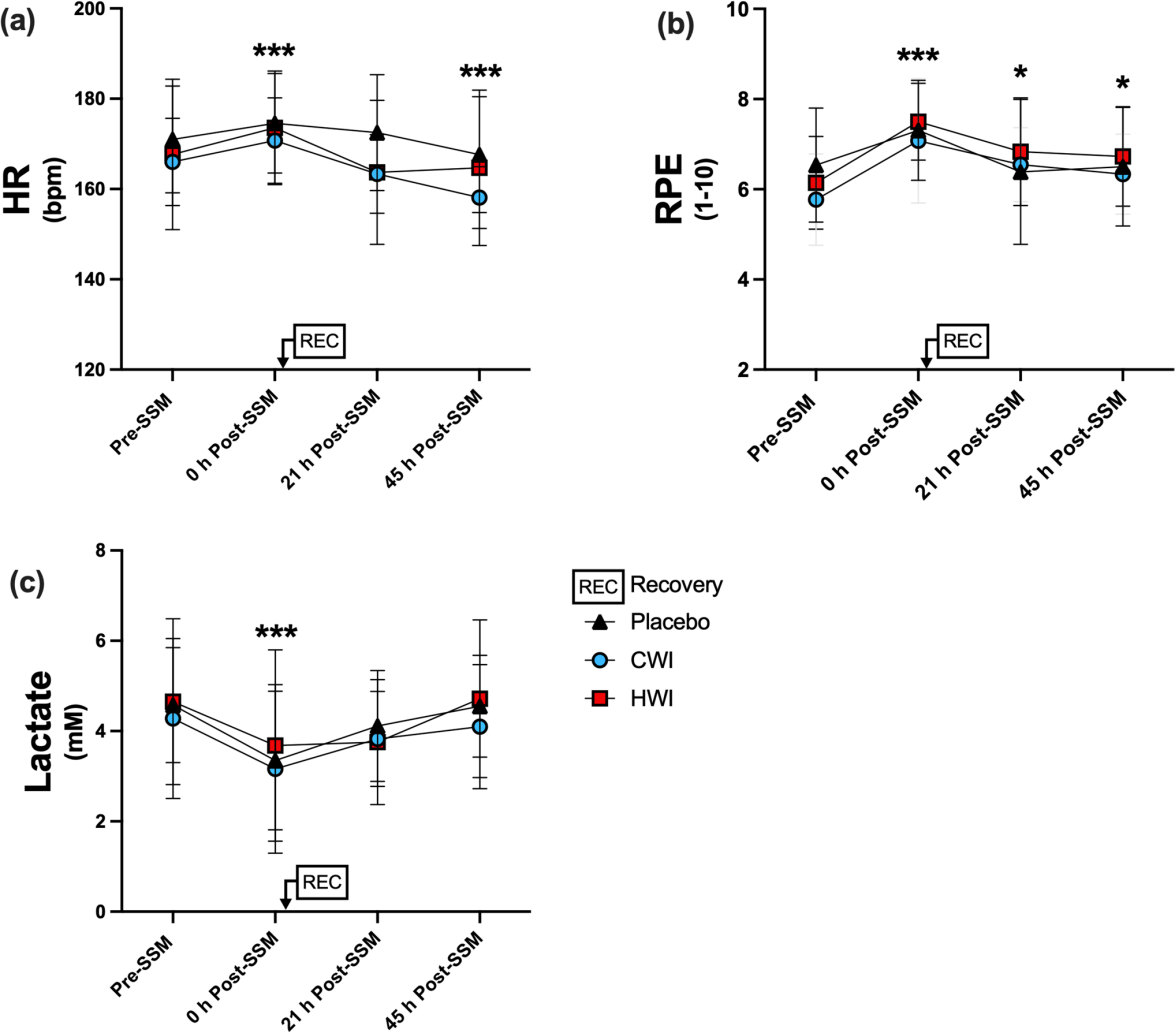
Heart rate (**a**), rating of perceived exertion (**b**) and lactate concentration (**c**) following the submaximal aerobic test (Part I). Data are presented as mean ± SD, *** p < 0.001 vs Pre-SSM. * p < 0.05 vs Pre-SSM. *CWI* cold-water immersion, *HR* heart rate, *HWI* hot-water immersion, *RPE* rating of perceived exertion, *SSM* simulated soccer match. A: Placebo, n = 12; CWI, n = 11; HWI, n = 12. B and C: Placebo, n = 13; CWI, n = 13; HWI n = 14

Compared to Pre-SSM, HR and RPE (assessed at the end of the submaximal aerobic test) were similarly elevated in the three groups directly after the SSM (Fig. 2a, b; time effect: p < 0.001). Recovery modality did not affect HR at 21 h and 45 h (interaction: p = 0.47), while a time effect was observed (p < 0.01). At 21 h, HR of the three groups had returned to Pre-SSM values, and at 45 h, HR was lower compared to Pre-SSM (p < 0.001). Recovery modality did not affect RPE at 21 h and 45 h (interaction: p = 0.45), while a time effect was found (p < 0.05). RPE across the three groups remained higher at 21 h and 45 h compared to Pre-SSM (p < 0.05). bLac was similarly decreased across the three groups immediately after the SSM (Fig. 2c; time effect: p < 0.01). It was neither affected by the recovery modality (interaction: p = 0.86) nor by time (p = 0.08) during the 21–45 h Post-SSM period.

#### Sprint test

**Fig. 3:**
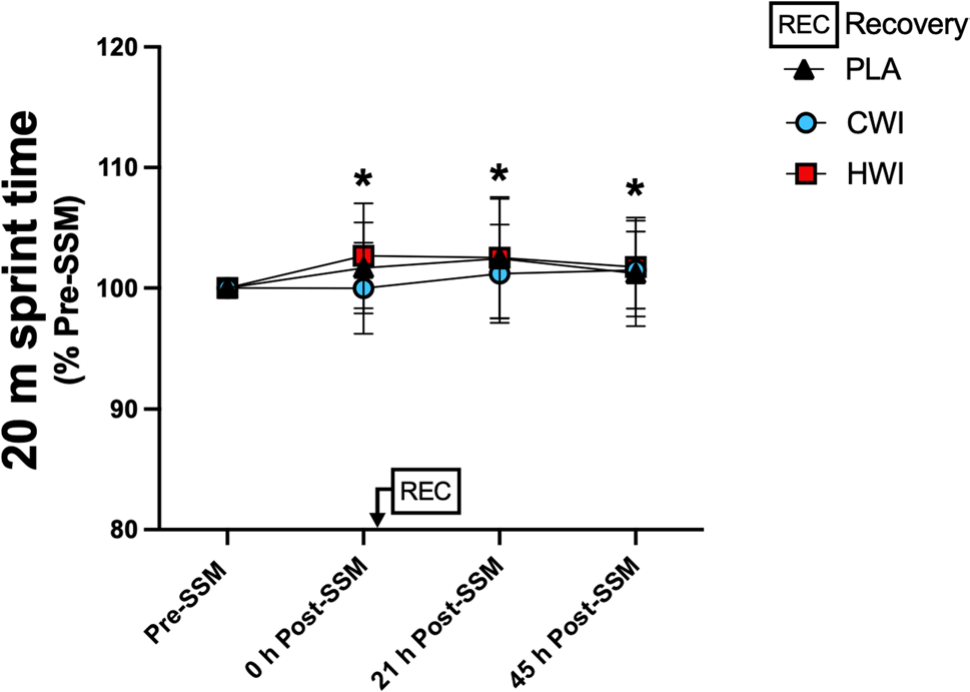
20 m sprint time (Part I). Data are presented as mean ± SD, * p < 0.05 vs Pre-SSM. *CWI* cold-water immersion, *HWI* hot-water immersion. *SSM* simulated soccer match. Placebo, n = 13; CWI, n = 13; HWI, n = 14

Directly after the SSM, 20 m sprint time was similarly increased by ~ 1.5% in all groups compared to pre-SSM (Fig. 3; time effect: p < 0.05). No effect of the recovery modality was observed at 21 h and 45 h (interaction: p = 0.90). Sprint time did not return to Pre-SSM values at 21 h and 45 h (p < 0.05).

#### CMJ test

**Fig. 4:**
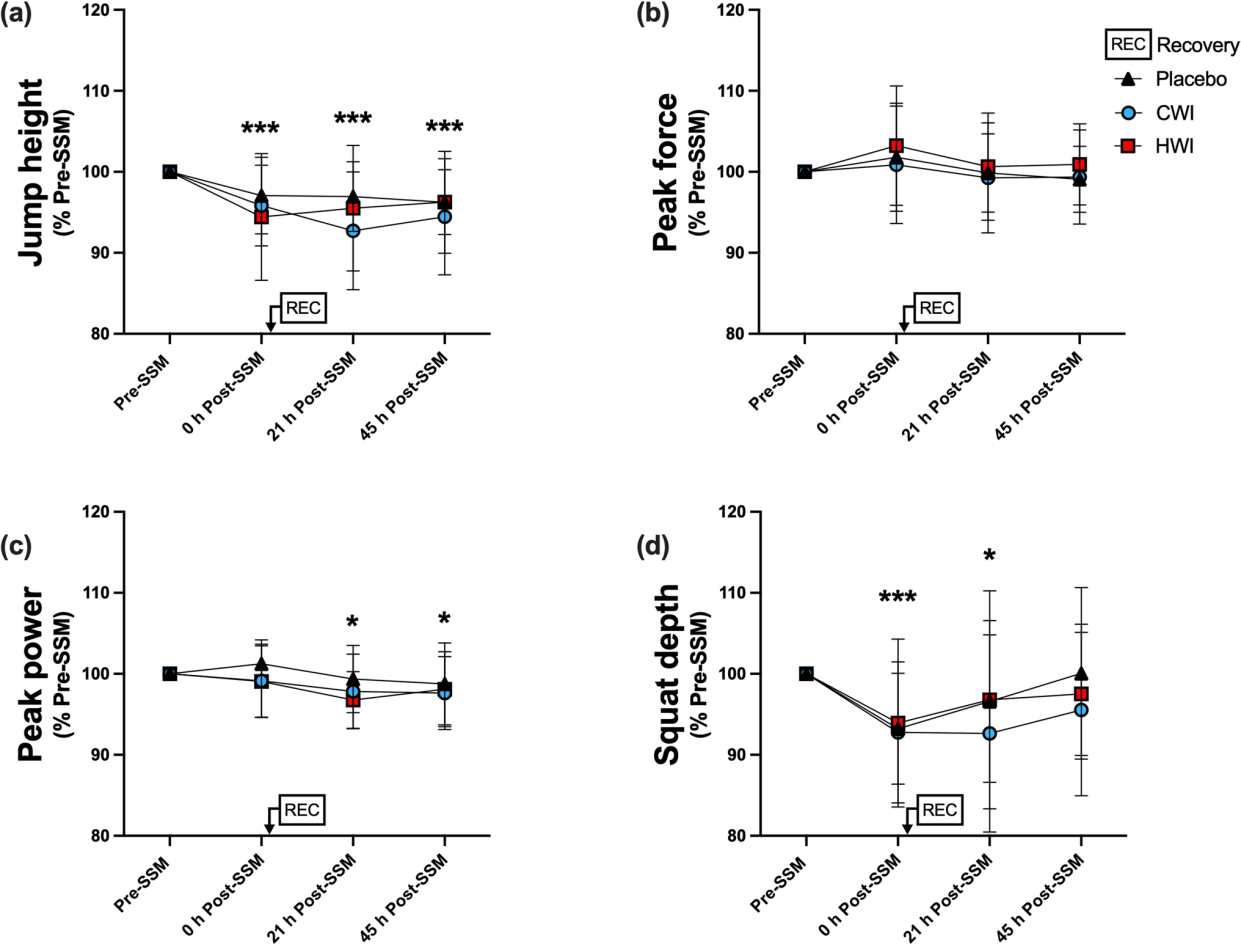
Jump height (**a**), Peak force (**b**), Peak power (**c**) and Squat depth (**d**) during the countermovement jump (Part I). Data are presented as mean ± SD, * p < 0.05 vs Pre-SSM. *** p < 0.001 vs Pre-SSM, *CMJ* counter movement jump, *CWI* cold-water immersion, *HWI* hot-water immersion, *SSM* simulated soccer match. Placebo, n = 13; CWI, n = 13; HWI, n = 14

CMJ height was impaired by ~ 4% in all groups directly after the SSM (Fig. 4a; time effect: p < 0.001). Recovery modality did not affect CMJ height at 21 h and 45 h (interaction, p = 0.66), while a time effect was observed (p < 0.001) such as CMJ height remained ~ 5% lower at both 21 h and 45 h after the SSM compared to Pre-SSM across the groups (p < 0.001). Peak force was not affected by the experimental conditions (Fig. 4b). Peak power was not affected directly following the SSM (Fig. 4c; time effect: p = 0.77). It was however similarly impaired by ~ 2% at 21 h and 45 h Post-SSM in all groups (p < 0.05). Directly following the SSM, CMJ squat depth was similarly decreased by ~ 7% across the groups (Fig. 4d; time effect: p < 0.001). During the 21–45 h Post-SSM period, it was affected by time (p < 0.05), but not by the recovery modality (interaction: p = 0.75). CMJ squat depth remained decreased by ~ 5% at 21 h (p < 0.05), while it was no longer different compared to Pre-SSM at 45 h Post-SSM.

#### Strength test

**Fig. 5:**
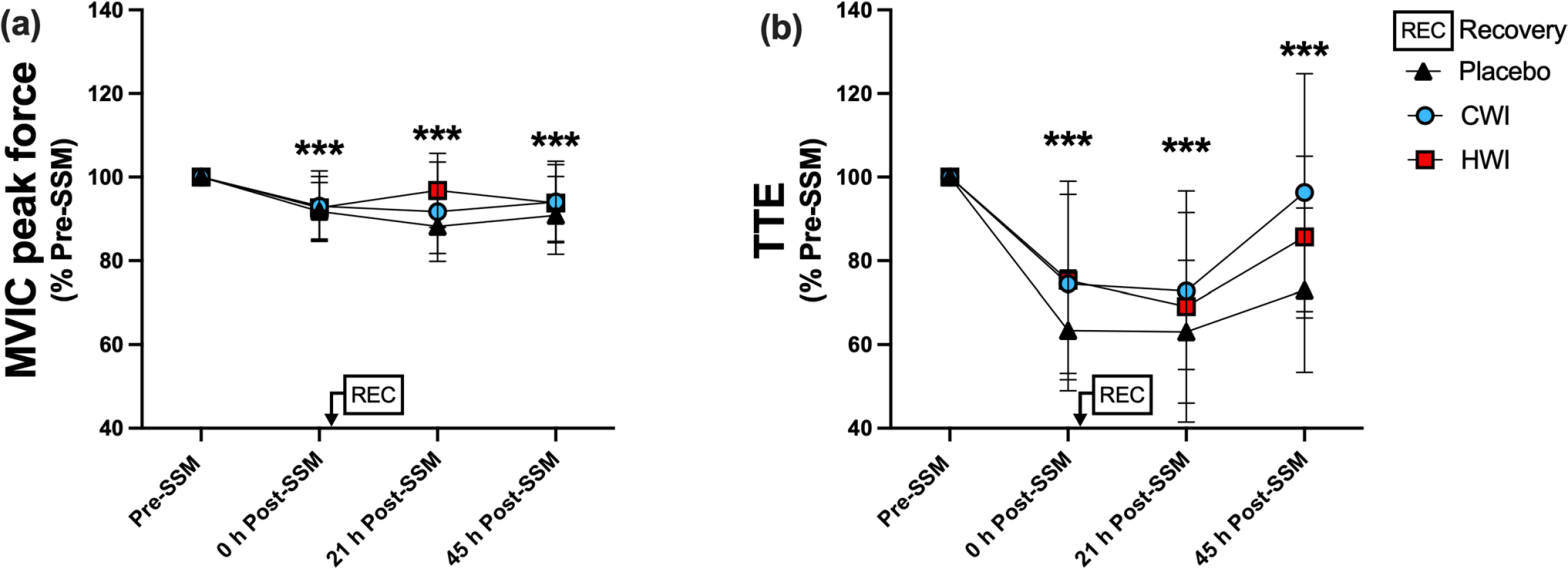
Strength of the knee extensors: Maximal voluntary isometric contraction (MVIC) peak force (**a**) and Time to exhaustion (**b**) (Part I). Data are presented as mean ± SD, *** p < 0.001 vs Pre-SSM. *CWI* cold-water immersion, *HWI* hot-water immersion, *SSM* simulated soccer match. Placebo, n = 13; CWI, n = 13; HWI, n = 14. B: Time to exhaustion (TTE) at 60% MVIC, Placebo, n = 13; CWI, n = 12; HWI n = 14

Directly following the SSM, MVIC peak force was similarly impaired by ~ 9% in all groups (Fig. 5a; time effect: p < 0.001). During the 21–45 h Post-SSM period, it was not affected by the recovery modality (interaction: p = 0.13), while a time effect was observed (p < 0.001). MVIC peak force across the groups remained impaired by ~ 7% at 21 h and 45 h Post-SSM (p < 0.001). Directly after the SSM, TTE was similarly reduced by ~ 30% in all groups compared to Pre-SSM (Fig. 5b; time effect: p < 0.001). During the 21–45 h Post-SSM period, TTE was affected by time (p < 0.001), but not by the recovery modality (interaction: p = 0.18). Overall, it remained impaired by ~ 30% and by ~ 15% at 21 h and 45 h Post-SSM, respectively (p < 0.001).

### Part II: Training adaptations over the 15-week intervention

#### Adherence

Over the 15 weeks, there were no between-groups differences in the number of soccer sessions (training and matches) (placebo: 64.3 ± 2.3, CWI: 62.9 ± 6.6, HWI: 62.8 ± 2.8 sessions, p = 0.80) and the number of strength sessions performed (placebo: 10.5 ± 1.0, CWI: 10.4 ± 2.1, HWI: 9.2 ± 2.3 sessions, p = 0.42). There was no difference between the number of recovery sessions performed by the groups (placebo: 26.0 ± 3.4; CWI: 26.0 ± 3.3; HWI: 27.2 ± 2.7 sessions, p = 0.76).

#### Height and body composition

Over the 15 weeks, the participants’ height increased more in the placebo and HWI (~ 1 cm, p < 0.05) compared to the CWI (~ 0.5 cm, p = 0.053) (Table 2; interaction: p < 0.05).

Body mass and fat-free mass increased similarly across all groups (~ 2 kg) over the 15 weeks (time effect: p < 0.001). For body fat percentage, a recovery modality effect was observed (p < 0.05), without any time effect or interaction.

#### Battery of physical performance tests

HR, RPE and bLac immediately after the submaximal aerobic test were not affected by the experimental conditions (Table 2).

For 20 m sprint time, there was an effect of time (p < 0.05), without any significant interaction (p = 0.07). The sprint time of the placebo and HWI groups decreased by 2.1 ± 2.1% and 1.4 ± 0.9%, respectively, while the sprint time in the CWI group remained relatively unchanged (+ 0.6 ± 2.5%) (Table 2).

For CMJ performance, there was a time effect for jump height (p < 0.05), with all three groups showing reduced jump heights after the 15 week intervention (Table 2; placebo: −1.8 ± 3.0%, CWI: −5.5 ± 6.6%, HWI: −2.7 ± 5.1%). Absolute and relative peak force were improved after the 15-week intervention in all groups (time effect: p < 0.05) (Absolute force: placebo: + 10.8 ± 6.9%, CWI: + 6.8 ± 9.3%, HWI: + 8.5 ± 12.5%). Absolute and relative peak power were not affected by time. Squat depth was slightly but not significantly reduced following the 15 week intervention (time effect: p = 0.058). No group effect or interaction was observed for these parameters.

Absolute and relative MVIC peak force were increased in all groups following the intervention (Table 2; time effect: p < 0.001) (Absolute peak force: placebo: + 24.0 ± 14.1%, CWI: + 11.5 ± 10.8%, HWI: + 18.2 ± 14.3%). For TTE, a time effect was observed, where all groups increased (placebo: + 13.3 ± 29.0%, CWI: + 19.8 ± 41.2%, HWI: + 46.6 ± 36.7%). No group effect or interaction was observed for these parameters.

## Discussion

The first aim of the present study was to compare the effect of post-exercise CWI, HWI and placebo on the recovery of physical performance in national level youth soccer players. Here we showed that all three modalities did not differently affect recovery of submaximal aerobic performance, 20 m sprint performance, CMJ performance, and maximal and submaximal strength 21 h–45 h after a SSM. The second aim was to investigate whether the repeated use of these post-exercise recovery modalities has an impact on training adaptations over a 15 week period. We did not observe any significant effect of the recovery modalities on body composition and on the development of physical performance over time.

### Part I

To our knowledge, this is the first study to compare the effects of CWI, HWI and placebo on the recovery of physical performance in soccer players. Our results indicate that the physical demands induced by our modified version of the Copenhagen soccer test (HR, RPE and bLac), were comparable to that observed during a soccer match (Bendiksen et al. 2012), which makes it suitable in our population. In addition, the performance (distance covered) and physical demands during the SSM were similar to those reported in elite youth (~ 18 year-old) soccer players during match play (Hunter et al. 2015; Pettersen et al. 2014), further confirming the high training status of our participants. Compared to Pre, the submaximal aerobic test following the SSM was more demanding, as illustrated by higher HR and RPE. Lower bLac values were likely due to the combined influence of increased bLac clearance stemming from elevated muscle blood flow, and possibly a shift in substrate utilization from carbohydrates to fat (Bendiksen et al. 2012). These parameters were not affected by the recovery modalities during the 45 h Post-SSM period. Our findings are in line with another study on soccer players showing no effect of CWI on Yo-Yo intermittent recovery test running distance at 24 h and 48 h, when undertaken in similar ambient conditions (< 9 °C) (Rupp et al. 2012). In contrast, CWI undertaken after soccer game (Rowsell et al. 2011), or endurance exercise (Brophy-Williams et al. 2011; Vaile et al. 2008) performed in warm or hot conditions has been shown to accelerate the recovery of endurance performance, likely due to accelerated reduction of core temperature and reduced cardiovascular strain (Vaile et al. 2008). To our knowledge, our study is the first to show that post-exercise HWI does not impact the recovery of submaximal running performance in soccer players (compared to CWI and placebo) after a SSM undertaken in relatively cool ambient conditions (< 9 °C). However, this conclusion might not apply after a soccer match performed in a hot environment where thermal stress is more intense and may impair recovery of endurance performance.

Our results indicate that the slight impairment in CMJ height directly after the SSM could be mainly related to a different execution pattern (as evidenced by decreased squat depth) (Sánchez-Sixto et al. 2018), due to Post-SSM fatigue, while it is likely due to a combination of reduced power and squat depth during the 21 h–45 h recovery period. Similarly to CMJ height, the slightly reduced performance in 20 m sprint after the SSM was not affected by recovery modality during the 45 h Post-SSM period. In soccer players, these findings are in line with a previous study (Coelho et al. 2021), where neither CWI nor heating (with Far-Infrared Emitting Ceramic Materials) improved recovery of these parameters. However, they are in contrast with other studies showing that CWI improves recovery of 20 m sprint (Bouchiba et al. 2022; Bouzid et al. 2018) and CMJ (Ascensão et al. 2011; Bouchiba et al. 2022; Bouzid et al. 2018; Pooley et al. 2020) performance over 48 h of recovery compared to control conditions. In addition, one of our main findings was that impairment in MVIC peak force seen immediately after and during the 45 h Post-SSM recovery period, did not differ between CWI, HWI and placebo. This is in contrast to other soccer studies, which have shown that CWI improves the restoration of MVIC peak force compared to control (thermoneutral water immersion) (Ascensão et al. 2011; Bouchiba et al. 2022; Bouzid et al. 2018). One originality of the current study was the assessment of fatigue resistance during a submaximal sustained contraction (TTE). A previous animal study indicated that cooling could suppress the recovery of submaximal force, while heating could have the opposite effect during the early recovery phase (< 2 h) in intact mouse single muscle fibers (Cheng et al. 2017). However, extrapolating and comparing these finding to our results is difficult due to substantial methodological differences between the two studies (e.g., ex vivo analyses on mouse single fibers vs. in vivo analyses in humans, exposure time to cooling and cooling modality). In our study, despite the large impairment in TTE (~ 30%), we provide evidence that neither cooling nor heating specifically alter the recovery of fatigue resistance during the later recovery phase (21–45 h post-exercise).

While discrepancies observed between our work and other studies may be partly explained by environmental conditions regarding submaximal aerobic test performance, another important factor to consider for all outcomes is the placebo effect (Beedie and Foad 2009). To date, the recovery benefits of CWI in soccer players have not been shown to be greater than any placebo effect (Nasser et al. 2023), which is in line with other studies on endurance (Wilson et al. 2018), resistance (Wilson et al. 2019), and repeated sprint (Broatch et al. 2014) exercises. Given our experimental design, all groups may have experienced recovery benefits to some extent, which were, however, not different between the groups. When compared to control conditions, similar enhancements in recovery of physical performance were observed following CWI and placebo, and were related to the belief in the recovery modalities in active males (Broatch et al. 2014) and semi-professional soccer players (Nasser et al. 2023). Our results show that the effect of CWI and HWI on recovery of physical performance are not greater than placebo.

### Part II

To our knowledge, this study was also the first to investigate the effect of post-exercise CWI, HWI and placebo over a prolonged (15 week) period of soccer training. The current intervention period was longer than that used in previous studies (4–12 weeks) in active individuals (Fyfe et al. 2019; Roberts et al. 2015) and athletes (Horgan et al. 2023; Méline et al. 2021). In our study, all groups grew (i.e. increased height, body mass and fat-free mass) during the intervention. These changes are likely due to the combination of biological maturation and training, and were not affected by recovery modalities. These findings are in line with two studies with shorter interventions (4 weeks) performed in athletes, where post-exercise HWI and CWI did not affect fat-free mass in short-track speed skaters (Méline et al. 2021) and rugby players (Horgan et al. 2023). These findings are also in line with other forms of heating in female team sport athletes, as post-exercise infrared sauna did not impact hypertrophy gains over a 6 week period (Ahokas et al. 2025). However, in active men, two weekly administrations of CWI post-resistance exercise attenuated the gain in muscle mass over 12 weeks (Roberts et al. 2015). Discrepancies between findings may be explained by differences in exercise type preceding the recovery treatment (resistance exercise vs sport-specific training), the intervention duration and frequency, the studied population (e.g. age, competitive levels and training loads), and the methods used to assess lean- and muscle mass (Magnetic resonance imagery, Dual X-ray absorptiometry, Bioelectrical impedance analysis). Our results on lean mass support other findings indicating that HWI does not further augment gains in muscle mass (Horgan et al. 2023; Méline et al. 2021).

Despite some limitations (lack of HR measurement in the CWI group and low sample size), our results suggest that the development of submaximal aerobic performance is not influenced by the recovery modalities. Indeed, a recent meta-analysis indicates that CWI (≤ 6 weeks) does not impact endurance training adaptations (Malta et al. 2021), which seems to be in line with our findings. On the other hand, four weeks of post-exercise HWI (20 min at 40 °C, 4 × per week) may improve VO_2_max in short-track speed skaters (VO_2_max gain observed in 5/6 athletes, p = 0.053) (Méline et al. 2021). In addition, 6 weeks of post-exercise HWI (15–30 min at ~ 40 °C, 2–3 × per week, whole body) improved intermittent running performance in Australian rules soccer players (Philp et al. 2022). While we did not observe any benefits of HWI on aerobic capacity, applying a greater heat stress (depth of immersion, exposure duration and frequency) may improve heat acclimation, ultimately leading to better endurance performance in a warm or hot environment.

Regarding the neuromuscular outcomes, 20 m sprint time, MVIC peak force, TTE, and CMJ peak force were improved in all groups after the intervention. As suggested above, in our population these changes may be attributed to the combination of biological maturation and training, considering that no significant effect of recovery modality was observed. Unexpectedly, CMJ height was slightly and similarly decreased in all groups after the intervention. The impairment of CMJ performance is likely due to alterations in jump execution patterns (as noted by a near significant reduction in squat depth of ~ 1–3.5 cm after the intervention, p = 0.058) despite the improvement of peak force (Sánchez-Sixto et al. 2018).

With regard to the development of CMJ performance, some studies have shown both a beneficial (Horgan et al. 2023; Tavares et al. 2019) and a detrimental (Fyfe et al. 2019) effect of CWI, while for HWI, a null (Méline et al. 2021), or detrimental effect (Horgan et al. 2023) has been shown. Our study provides new evidence suggesting that CWI and HWI do not significantly affect the development of CMJ performance. Furthermore, short interventions incorporating post-exercise CWI (≤ 8 weeks) are shown not to impact gains in maximal leg strength (Fröhlich et al. 2014; Fyfe et al. 2019; Wilson et al. 2021), while longer interventions (12 weeks) have been shown to mitigate such improvement (Roberts et al. 2015). To our knowledge, the only study on repeated post-exercise HWI (4 weeks) showed that this modality positively affected leg strength (Méline et al. 2021) in a mixed cohort of short-track speed skaters, while other forms of local heating (heat pads) do not confirm such benefits in healthy, untrained males (Stadnyk et al. 2018). Despite our long intervention, we show that CWI and HWI do not significantly influence leg strength gains in youth soccer players. In addition to the aforementioned factors, discrepancies between findings across the literature could be due to recovery protocols, neuromuscular outcomes measured, the inclusion of a placebo, and sample sizes. Notably, while no effect on individual performance indicators was observed, when viewed together, a pattern seems to emerge where CWI elicited the lowest development of neuromuscular performance (e.g. CMJ peak force, MVIC peak force, 20 m sprint time) among the three recovery intervention modalities of our study. This observation should be interpreted with caution due to the limited sample size.

### Limitations

In our study, the recovery modalities were applied after the physical performance test battery following the SSM, instead of directly after it. This methodological choice was made to ensure that the SSM similarly induced fatigue across the groups. Although the participants were still exercising during the test battery (45 min), this delay may have affected our outcomes (Brophy-Williams et al. 2011). Furthermore, the collection of biological samples (specifically muscle micro-biopsies; see method section) may have slightly influenced physical performance. This effect was likely limited and comparable across the three groups and over the assessment period. In addition, the lack of a “true” control group (i.e. undertaking only passive rest) may undermine any recovery benefits shared across the three groups included in our study. This, together with the lack of belief questionnaires limits interpretations regarding the extent of any placebo effect (Broatch et al. 2014). Finally, conclusions from Part II should be taken with caution given the limited sample size and HR data.

### Conclusion and perspectives

For recovery following a single soccer match or session, our results support the view that CWI and HWI are not more effective than a placebo, at least when the exercise is not performed in a hot environment. Future studies should investigate whether similar findings are observed in warmer environments. Our results should also be considered in the context of male adolescent soccer players. Responses may differ in adult players, where greater levels of fatigue could necessitate enhanced recovery (Zafeiridis et al. 2005). Furthermore, future research could investigate whether our findings are similar in females, who display biological and morphological differences (Karastergiou et al. 2012). Our results suggest that, compared to a placebo sham laser treatment, repeated CWI and HWI do not augment adaptations to soccer training. Various placebo treatments have shown to be similarly effective as CWI and better than control conditions (Broatch et al. 2014). Therefore, for coaches and teams looking to implement recovery modalities after matches and training, it seems wise to consider a) the ambient conditions, and b) the combination of belief and comfort for athletes when choosing recovery modalities. Finally, future work will focus on the effect of these post-exercise recovery modalities on muscle damage, inflammatory and metabolic responses.

## Supplementary Information

Below is the link to the electronic supplementary material.

Supplementary file1 (PDF 546 KB)

## Data Availability

Data collected in this study are available upon reasonable request to the corresponding author.

## Abbreviations:

ANOVA: Analysis of variance
bLac: Blood lactate concentration
CMJ: Countermovement jump
CWI: Cold-water immersion
HR: Heart rate
HWI: Hot-water immersion
MVIC: Maximal voluntary isometric contraction
RPE: Rating of perceived exertion
SSM: Simulated soccer match
TTE: Knee extension time to exhaustion
