# Supplementary material for: Cold- and hot-water immersion are not more effective than placebo for the recovery of physical performance and training adaptations in national level soccer players
Source: Eur J Appl Physiol. 2025 Jun 11;125(11):3179–94. doi: 10.1007/s00421-025-05835-w (PMC12528220; doi:10.1007/s00421-025-05835-w)

Cold- and Hot-water immersion are not more effective than Placebo for recovery and training adaptations in national level football players

European Journal of Applied Physiology

Jannik Gustafsson[1]*, Diego Montiel-Rojas*, Mattias Romare, Elin Johansson, Mattias Folkesson, Marco Pernigoni, Anastasija Frolova, Marius Brazaitis, Tomas Venckunas, Elodie Ponsot, Thomas Chaillou, Peter Edholm

*co-first author

1 School of Health Sciences, Örebro University, Örebro, Sweden

Correspondence: Jannik Gustafsson (Jannik.gustafsson@oru.se) ORCiD: 0009-0006-5201-529X

Supplementary material

Fig. 1: Representative CMJ

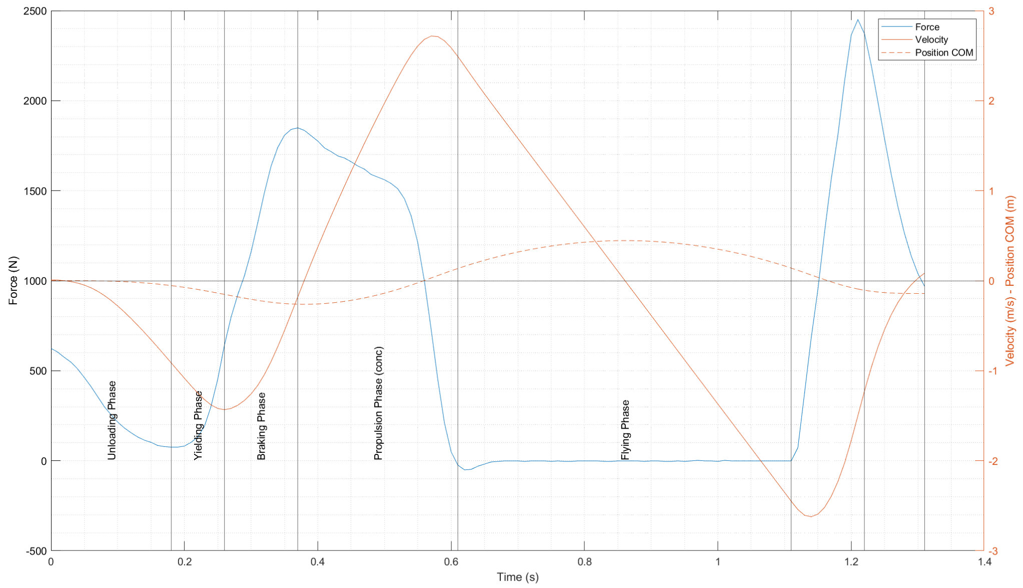

Fig. 2: Representative Knee extension time to exhaustion at 60% MVIC peak force

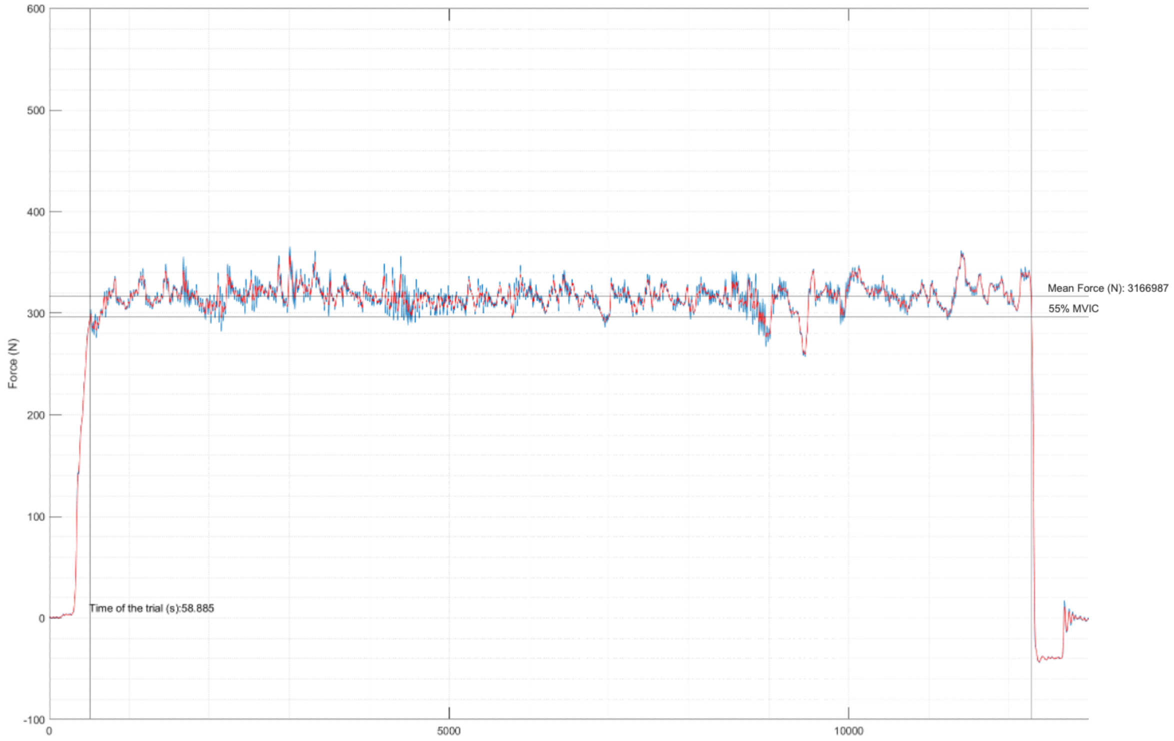

Supplement: Supplementary file 1 — Supplementary file1 (PDF 546 KB) [file 421_2025_5835_MOESM1_ESM.pdf]
